# Changes in Cerebrospinal Fluid Tau and β-Amyloid Levels in Diabetic and Prediabetic Patients: A Meta-Analysis

**DOI:** 10.3389/fnagi.2018.00271

**Published:** 2018-10-11

**Authors:** Yanhui Lu, Xinjun Jiang, Shuling Liu, Mingzi Li

**Affiliations:** School of Nursing, Peking University Health Science Center, Beijing, China

**Keywords:** Alzheimer's disease, diabetes, meta-analysis, β-amyloid, tau protein

## Abstract

Increased risks for Alzheimer's disease (AD) are a well-recognized consequence of diabetes, insulin resistance (IR), and hyperinsulinemia. Since cerebrospinal fluid (CSF) is surrounding the central nervous system, alterations of β-amyloid (Aβ) and tau protein in the CSF may be indicative of AD-type degenerations in the brain. Current laboratory diagnosis of AD uses three biomarkers in CSF: Aβ1-42, total tau (t-Tau), and phosphorylated tau (p-Tau). However, changes in these biomarkers in diabetic and prediabetic patients are scattered and variable in literature. Thus, we attempt to perform a systematical analysis of these available data. MEDLINE, EMBASE, the Cochrane Central database, China National Knowledge Infrastructure (CNKI), and Wanfang Data electronic databases were searched to gather published studies that have evaluated the AD-type biomarkers in the CSF of subjects with diabetes, IR, or hyperinsulinemia in comparison with respective controls. Overall analysis of the published data showed no significant differences in Aβ1-42, t-Tau, and p-Tau levels in the CSF between the (pre)diabetic subjects and controls. However, subgroup analysis suggested that (pre)diabetic conditions might accelerate decrease of Aβ1-42, but increase of t-Tau levels in the CSF of subjects with cognitive impairment, and the association with p-Tau in the CSF was stronger (*P* = 0.001) for diabetes than those of prediabetes (*P* = 0.61). Our analyses reveal that the relationship between (pre)diabetic conditions and AD-type biomarker status in the CSF was subjective to clinical characteristics.

## Introduction

Diabetes represents a group of metabolic disorders caused by impaired insulin signaling and function. In addition to elevated blood glucose and insulin resistance (IR) in peripheral tissues, diabetes leads to a number of complications and comorbidities including cognitive dysfunction (American Diabetes Association, 2013). In 1950, the term “diabetic encephalopathy” was first employed to describe brain dysfunction in patients with diabetes. From then on, mounting evidences from longitudinal and cross-sectional studies as well as biological research have demonstrated a significant association between diabetes and increased risks of multiple-domain cognitive decline and even dementia (McCrimmon et al., 2012). Several excellent meta-analyses have described that diabetes results in mild-to-moderate deficits in multiple cognitive domains, especially processing speed, attention, memory, and executive function (Monette et al., 2014; Palta et al., 2014; Sadanand et al., 2016). Furthermore, other meta-analyses have found that diabetes is a significant risk factor for mild cognitive impairment (MCI) and incident dementia including Alzheimer's disease (AD), vascular dementia (VD), and any dementia (Cheng et al., 2012). Dementia is an age-related chronic and progressive disorder in late life. A characteristic feature of dementia is the deterioration of cognitive function beyond normal aging process. Approximately, 4.6 million new cases of dementia are estimated to occur globally every year (Ferri et al., 2005). The incidence of dementia and the number of individuals living with dementia are expected to be doubled in the next 20 years, which will cause enormous social and economic burdens (Tariq and Barber, 2017). The AD is the most common type of dementia, characterized by two pathological hallmarks including formation of senile plaques (SPs) by extracellular deposits of β-amyloid (Aβ) and intracellular neurofibrillary tangles (NFT) from aggregated hyperphosphorylated Tau proteins in the brain (Jack et al., 2013), which are associated with elevated levels of phosphorylated Tau (p-Tau) and decreased levels of the nonsoluble Aβ1-42 from abnormal cleavage of Aβ in the cerebrospinal fluid (CSF) (Blennow and Hampel, 2003). An updated meta-analysis of cohort studies has concluded that the risk of AD is significantly higher in diabetic patients than control subjects, especially in Eastern populations [relative risk (RR):1.62, 95% confidence interval (CI): 1.49–1.75] (Zhang et al., 2017).

While AD was not identified as one of the hallmark comorbidities of diabetes, epidemiological and biological evidences have suggested a link between these two disorders. Over the past 30 years, investigations on the potential mechanisms and pathways in this regards have grown rapidly. There have been increasing numbers of studies describing vascular, metabolic, and neuroendocrine contributions to AD (Kodl and Seaquist, 2008; Banks et al., 2012). With regards to characterizing AD pathology, phosphorylated Tau (p-Tau) and total Tau (t-Tau) are elevated, while Aβ1-42 is decreased in the CSF. Elevations of t-Tau and p-Tau in the CSF are biomarkers of tauopathy in AD and they correlated well with intracerebral AD pathology, while decreases of Aβ1-42 in the CSF are inversely proportional to amyloid in the brain (Reitz, 2012). There is also an increasing evidence that patients with diabetes shared commonality with neuropathology in AD (Matioli and Nitrini, 2015). Moreover, the risk of AD has also been shown to be increased in prediabetes individuals with high fasting glucose and/or impaired glucose tolerance due to IR and simultaneous exposure to abnormally high levels of insulin persisting for extended periods of time (hyperinsulinemia) (Roriz-Filhom et al., 2009). The IR and hyperinsulinemia are also reported to be linked with the pathological features of AD (Luchsinger et al., 2004; Westwood et al., 2017).

However, there have been reports with conflicting results (Moran et al., 2015). Apparently, the association between diabetes or prediabetes and AD-like pathology in the CSF is still a subject of controversy. Thus, we collected data from published case–control studies of diabetes, prediabetes, and CSF biomarkers, and performed a meta-analysis to help clarify the association between diabetes or prediabetes and CSF biomarkers of neurodegeneration implicated in the development of AD.

## Methods

### Data source and search

The Cochrane Library, Medline, EMBASE, China National Knowledge Infrastructure (CNKI), and Wanfang Data electronic databases (from their inception to December 1, 2017) were searched to identify human studies published in English and Chinese. The search terms and key words included “diabetes,” “Alzheimer,” “insulin,” “cerebrospinal fluid,” “amyloid,” “tau,” and “dementia.” These key words were combined with type 2 diabetes (T2D), type 2 diabetes mellitus (T2DM), type 1 diabetes (T1D), type 1 diabetes mellitus (T2DM), IR, hyperinsulinemia, hyperglycemia, glucose, glycemia, impaired glycemia, ApoE, duration, complications, and treatment modality to locate studies on (pre)diabetes and associated variables. Reference lists from relevant original and review articles were also screened and potentially relevant papers were retrieved and assessed in accordance with the selection criteria. Citations and abstracts of all the studies have been checked to prevent duplications.

### Study selection

The protocol for selecting an eligible study was based on inclusion and exclusion criteria being screened at two levels. Firstly, the title and abstract of the paper were screened to identify whether the study fulfilled the inclusion criteria. Next, the initially selected papers were retrieved for full text by two independent reviewers to establish the final eligibility of the articles. Disagreements were adjudicated by a third reviewer based on the full text.

Studies in this meta-analysis were assessed for eligibility by fulfillment of the criteria of the population, intervention, comparison, outcomes, and setting (PICOS) question format and the details of inclusion and exclusion were as follows.

#### Population

We included studies with adults diagnosed with (pre)diabetes, including T1DM, T2DM, IR, hyperinsulinemia, hyperglycemia, or impaired glycemia, according to the criteria generally accepted. Studies without a non(pre)diabetic comparison group were excluded.

#### Intervention

We included only case–control studies; interventions were not taken into consideration.

#### Comparison

We included studies with comparison with (pre)diabetics or non(pre)diabetics, which allocated (pre)diabetic patients according to criteria of diagnoses. Review articles, case reports, commentaries, clinical trials, or letters were excluded.

#### Outcome

Studies were included if they measured at least one of the outcomes of CSF levels of AD-type biomarkers and presented original data on CSF biomarkers to permit effect size calculations (means, SD, SE, 95%CI or SEM).

#### Setting: case–control studies

**Figure 1:**
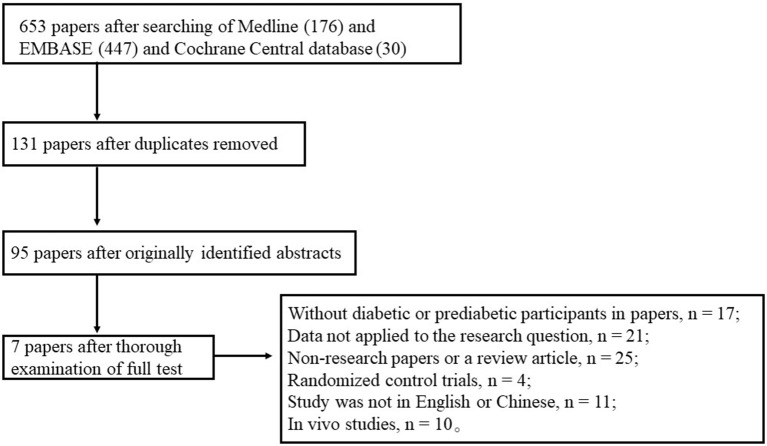
Flow diagram of selection of studies focusing on levels of Aβ or tau proteins in the CSF of diabetic subjects.

Our database searches resulted in 653 articles. Initial screening yielded 131 studies for title or abstract review, and 95 of these articles underwent full text review. After following a thorough examination for this meta-analysis, we excluded 87 of the 95 studies (without diabetic or prediabetic participants in papers, *n* = 17; data not applied to the research question, *n* = 21; nonresearch papers or review articles, *n* = 25; randomized control trials, *n* = 4; study was not in English or Chinese, *n* = 11; or *in vivo* studies, *n* = 10). The remaining 7 articles were included in this analysis and a flow diagram of the study selection is presented in Figure 1.

### Quality assessment

The quality of all the included studies was appraised using the checklists of the Newcastle–Ottawa scale (Table 1). These checklists included selection (cases definition, representativeness of cases, selection of controls and definition of controls with 4 points), comparability (age and gender and additional factors with 2 points), and exposure/outcome (ascertainment of exposure, same method for case and control and nonresponse rate with 3 points). Quality assessment was according to the guidelines for reporting meta-analyses of observational studies. Scores were awarded up to 9 scores, with the highest quality and the final score of at least 6 points indicating adequate quality that can be included in our analysis (Bashashati et al., 2017; Garcez et al., 2018; Guo et al., 2018). Two of the authors independently evaluated potentially acceptable (included) articles in accordance with these criteria, and discrepancies were dealt with by discussion.

**Table 1:** Quality assessment of included studies using the Newcastle–Ottawa scale.

**Reference**	**Selection**				**Comparability**		**Exposure**			**Score**
	**Cases definition**	**Representativeness of cases**	**Selection of controls**	**Definition of controls**	**Age and gender**	**Additional factors**	**Ascertainment of exposure**	**Same method for case and control**	**Non-response rate**	
Moran et al., 2015	*			*		*	*	*	*	6
Ouwens et al., 2014	*	*		*	*	*	*	*	*	8
Li et al., 2018	*	*		*			*	*	*	6
Morris et al., 2014	*	*		*	*	*	*	*	*	8
Westwood et al., 2017	*	*	*	*		*	*	*	*	8
Xijiu, 2005	*			*	*	*	*	*	*	7
Lu et al., 2011	*		*	*	*	*	*	*	*	8

### Data extraction and conversion

Important details regarding the participants, methods, and measurements were extracted from the selected studies and summarized in Table 2. The elements of the checklist included (1) details of publication including first author's name and publication year; (2) characteristics of the participants including sample size, age, gender, categorization; (3) CSF levels of Aβ, p-Tau, or t-Tau; and (4) methods of measurement.

**Table 2:** Extracted and summarized details on subjects, methods, and measurements of the included studies.

**Reference**	**Participants categorization**		**CSF sample collection**	**Measurement**	**Biomarkers**	**ApoE genotype**	**Years of diabetic condition**	**Drug taking**	**Recruitment**	**Other factors that may affect cognitive function**
	**Cases**	**Controls**								
Moran et al., 2015	*n* = 124(T2DM) Age: 75.5 ± 6.2 Male: 68.5%	*n* = 692 Age: 74.1 ± 7.0 Male: 56.2%	Lumbar puncture was performed with a 20- or 24-gauge spinal needle	Fasting CSF was collected and analyzed using a Luminex platform (Luminex Corporation, Austin, TX) with an Innogenetics immunoassay kit (INNO-BIA AlzBio3; Ghent, Belgium) that included monoclonal antibodies for Aβ142, T-Tau and p-Tau181 (pg/mL).	Aβ1-42 t-Tau p-Tau181	–	–	76 T2DM cases with oral diabetes medications, 10 with insulin use and 5 with insulin and oral agent	A longitudinal multicenter study entitled Alzheimer's Disease Neuroimaging Initiative (ADNI) from over 50 sites across the United States and Canada	42 T2DM cases and 279 controls were smokers; 59 T2DM cases and 338 controls were MCI and 27 T2DM cases and 164 controls were AD dementia.
Ouwens et al., 2014	*n* = 37 (T1DM) Age: 42.6 ± 8.8 Male: 40.5%	*n* = 15 Age: 39.9 ± 11.3 Male: 40.0%	CSF was collected via a lumbar puncture in the L3/L4 or L4/L5 intervertebral space, using a 25-gauge needle.	Commercial ELISAs were used to determine the levels of Aβ42, Tau, pTau (Thr181) (all from Innogenetics, Ghent, Belgium)	Aβ1-42 t-Tau p-Tau181	13 T1DM cases and 7 controls were ApoE ε4 carriers	For type 1 diabetic patients a disease duration of at least 10 years	–	T1DM patients were recruited from the departments of Endocrinology and Ophthalmology of the VU University Medical Center, Amsterdam, the Netherlands, the department of Internal Medicine, Groene Hart Hospital, Gouda, the Netherlands, and by advertisements in diabetes magazines and a national newspaper.	Participants were excluded if they had a BMI above 35 kg/m2, current use of drugs affecting cerebral functioning, alcohol abuse (more than 20 g of alcohol per day), psychiatric disorders, anemia, thyroid dysfunction, use of glucocorticoids, hepatitis, stroke, severe head trauma, epilepsy.
Li et al., 2018	*n* = 77 (T2DM) Age: 70.48 ± 6.71 Male: 59.7%	*n* = 735 Age: 72.17 ± 7.37 Male: 52.2%	CSF samples were collected by following standard procedures stated in the ADNI protocols. In brief, all CSF samples were collected from the participants after at least a 6-hour fasting period.	AD biomarkers including Ab1-42 were measured at the ADNI Biomarkers Core located at the University of Pennsylvania. The CSF samples were analyzed by following storing, shipping, and testing procedures and with parallel strict quality control steps.	Aβ1-42	39 T2DM cases and 327 controls were ApoE ε4 carriers	–	–	A longitudinal multicenter study entitled Alzheimer's Disease Neuroimaging Initiative (ADNI) from over 50 sites across the United States and Canada	54 T2DM cases and 493 controls were MCI and 2 T2DM cases and 7 controls were AD dementia.
Morris et al., 2014	*n* = 97 (Impaired glycemia) Age: 75.8 ± 6.5 Male: 69.1%	*n* = 167 Age: 74.1 ± 7.6 Male: 58.7%	–	Cerebrospinal fluid was analyzed by ADNI for amyloid-b (Ab) and phosphorylated-tau(p-Tau) using the multiplex xMAP Luminex platform (Luminex Corporation, Austin, TX)	Aβ1-42 p-Tau	58 impaired glycemia cases and 87 normoglycemic controls were ApoE ε4 carriers	–	–	A longitudinal multicenter study entitled Alzheimer's Disease Neuroimaging Initiative (ADNI) from over 50 sites across the United States and Canada	–
Westwood et al., 2017	*n* = 28 (Insulin resistant) Age: 63 ± 4	*n* = 30 Age: 62 ± 5	CSF samples were collected by lumbar puncture at the L3/L4 or L4/L5 interspace. All samples were obtained in the morning according to a standard protocol	CSF concentrations of the 42 amino acid form of amyloid β (Aβ1–42), t-Tau and p-Tau were measured using sandwich ELISAs (INNOTEST; Fujirebio, Ghent, Belgium).	Aβ1–42 t-Tau p-Tau	10 insulin resistant cases and 13 insulin nonresistant controls were ApoE ε4 carriers	–	–	Participants were selected from the Metabolic Syndrome in Men (METSIM) study performed at the University of Eastern Finland, Kuopio, Finland	–
Xijiu, 2005	*n* = 21 (cognitive handicap patients with cerebral infarction and T2DM) Age: 64.89 ± 5.9 Male: 57.1%	*n* = 21 (cognitive handicap and cerebral infarction patients without T2DM) Age: 64.94 ± 2.1 Male: 57.1%	The cerebrospinal fluid was collected by polypropylene tube, centrifuged (2000 g, 10 min) within 4 h after lumbar puncture, and stored in a refrigerator at −20°C for use.	In this study, the levels of CSF biomarkers were tested by ELISA using Innotest h tau-Ag and Inno test β-amyloid (42), (Innogenetics, Belgium).	Aβ1–42 t-Tau	–	5.1–8.1	–	Memory clinics	Both case and control groups were patients with cognitive handicap and cerebral infarction
Lu et al., 2011	*n* = 23 (MCI patients with insulin resistant)	*n* = 20 (MCI patients without insulin resistant)		ELISA	Aβ1–42 t-Tau	–	–	–	Memory clinics	Both case and control groups were MCI patients
	Age:65.40 ± 8.73, Male: 62.82%								

### Statistical analysis

Data were compiled as summary statistics (N, mean, and SD) and then pooled by using an inverse-variance method. Heterogeneity among the studies was evaluated using Cochran's Chi-Squared test for homogeneity (Chi2) and estimated by calculating the I2. Random-effects meta-analyses were performed using Review Manager Version 5.3 to generate summary values, since heterogeneity was invariably high. Forest plots were presented and the results were determined to be significant when *P* < 0.05. Heterogeneity across studies was assessed according to *I*^2^ statistics, which was categorized as low (< 40%), moderate (40–75%), or high (>75%) to indicate the percentage of variance owing to study heterogeneity (Islam et al., 2017). Four independent subgroup analyses were conducted as follows: (i) diabetes vs. prediabetes (e.g., IR, hyperinsulinemia, hyperglycemia, or impaired glycemia); (ii) mean age of group below 65 years old vs. above 65 years old; (iii) subjects recruited through memory clinics vs. not recruited through memory clinics; (iv) studies with high quality (total score of NOS scale was at least 8) vs. studies with fair quality (total score of NOS scale was 5 to 7) (Alobaidi et al., 2018).

## Results

### Study characteristics and heterogeneity

**Figure 2:**
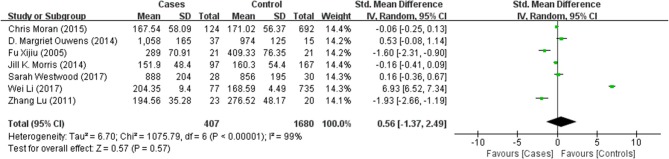
Forest plot comparing CSF Aβ1-42 levels in subjects with (pre)diabetes and controls.

**Figure 3:**
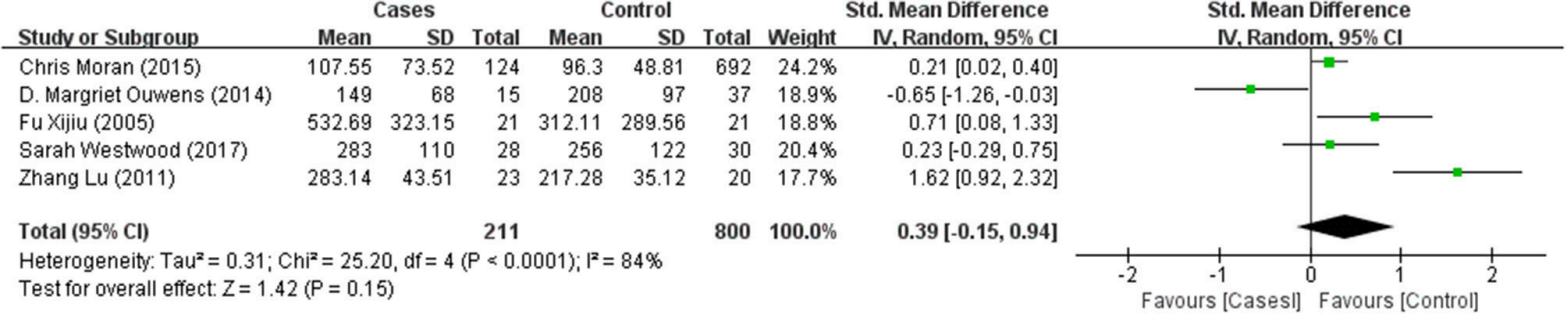
Forest plot comparing CSF t-Tau levels in subjects with (pre)diabetes and controls.

**Figure 4:**
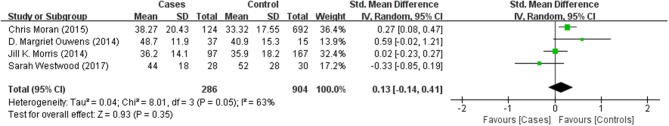
Forest plot comparing CSF p-Tau levels in subjects with (pre)diabetes and controls.

Tables 1, 2 summarize the 7 studies included in this meta-analysis (Xijiu, 2005; Lu et al., 2011; Morris et al., 2014; Ouwens et al., 2014; Moran et al., 2015; Westwood et al., 2017; Li et al., 2018). All of these studies were identified to be of high or fair quality by using the Newcastle–Ottawa scale (Table 1). The diagnosis of diabetes or prediabetes was made according to the American Diabetes Association (ADA) guidelines and/or the reference. Heterogeneity among these studies was assessed (cases vs. controls: Figure 2, Aβ1-42, Chi^2^ = 1075.79, *I*^*2*^ = 99%; Figure 3, t-Tau, Chi^2^ = 25.20, *I*^*2*^ = 84%; Figure 4, p-Tau, Chi^2^ = 8.01, *I*^*2*^ = 63%). Since the heterogeneity ranged from moderate to high, the random-effects meta-analyses were employed.

Seven studies reporting Aβ levels were included in this meta-analysis, including 407 subjects with (pre)diabetes and 1,680 controls. The Aβ levels in the CSF were not different between the (pre)diabetic subjects and controls, with an effect size of 0.56 (95%CI: −1.37, 2.49, *P* = 0.57, Figure 2).

T-Tau in (pre)diabetic subjects was reported in 5 studies including 211 (pre)diabetes subjects and 800 controls. Cumulatively, t-Tau was not significantly increased in the CSF of (pre)diabetes subjects compared with that of controls, with an effect size of 0.39 (95%CI: −0.15, 0.94, *P* = 0.15, Figure 3).

Four studies reported p-Tau in the CSF of 286 (pre)diabetic and 904 control subjects. There was no significant difference in the level between the two groups (effect size 0.13, 95% CI: −0.14, 0.41, *P* = 0.35, Figure 4).

**Figure 5:**
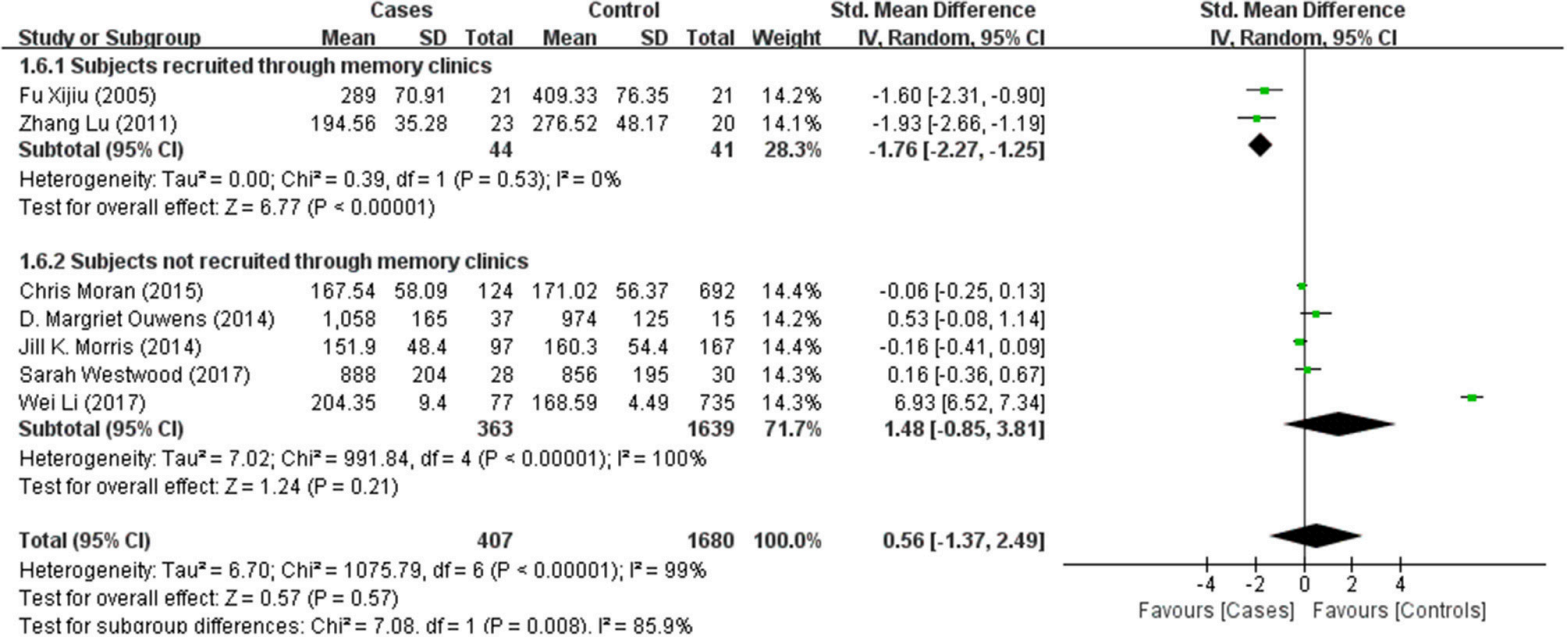
Subgroup analysis of CSF Aβ1-42 levels in subjects recruited in memory clinics or not.

**Figure 6:**
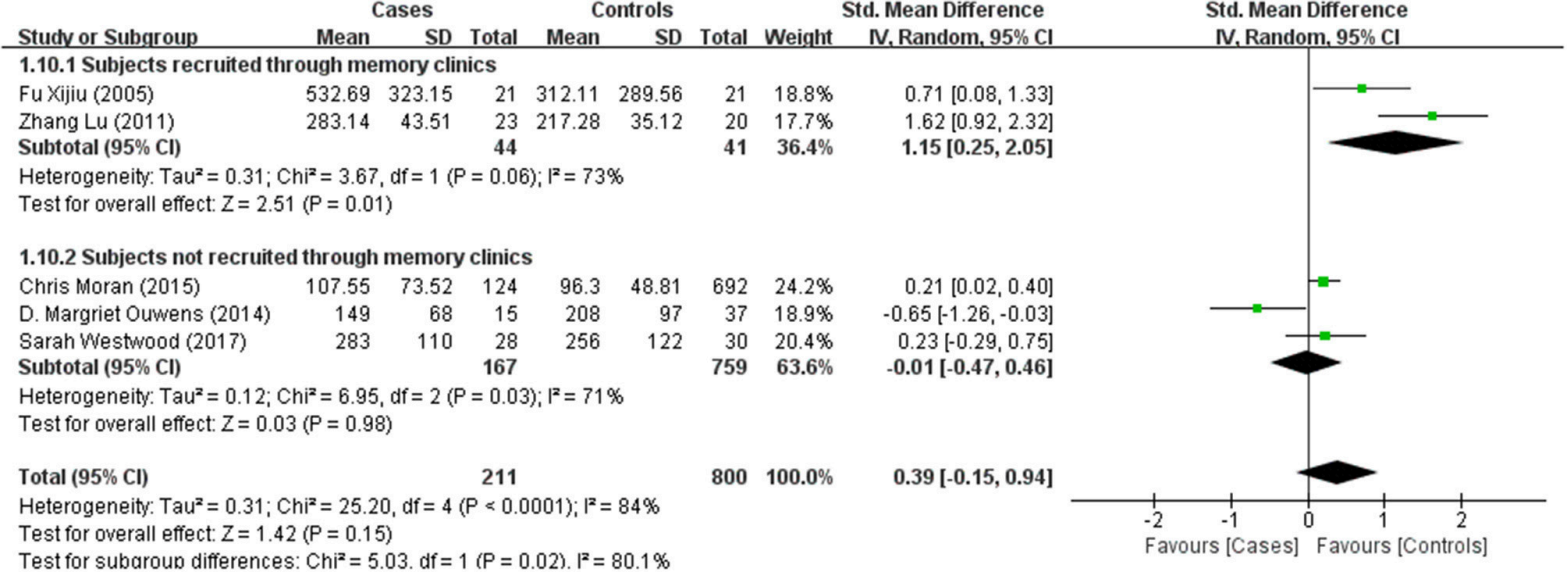
Subgroup analysis of CSF t-Tau levels in subjects recruited in memory clinics or not.

**Figure 7:**
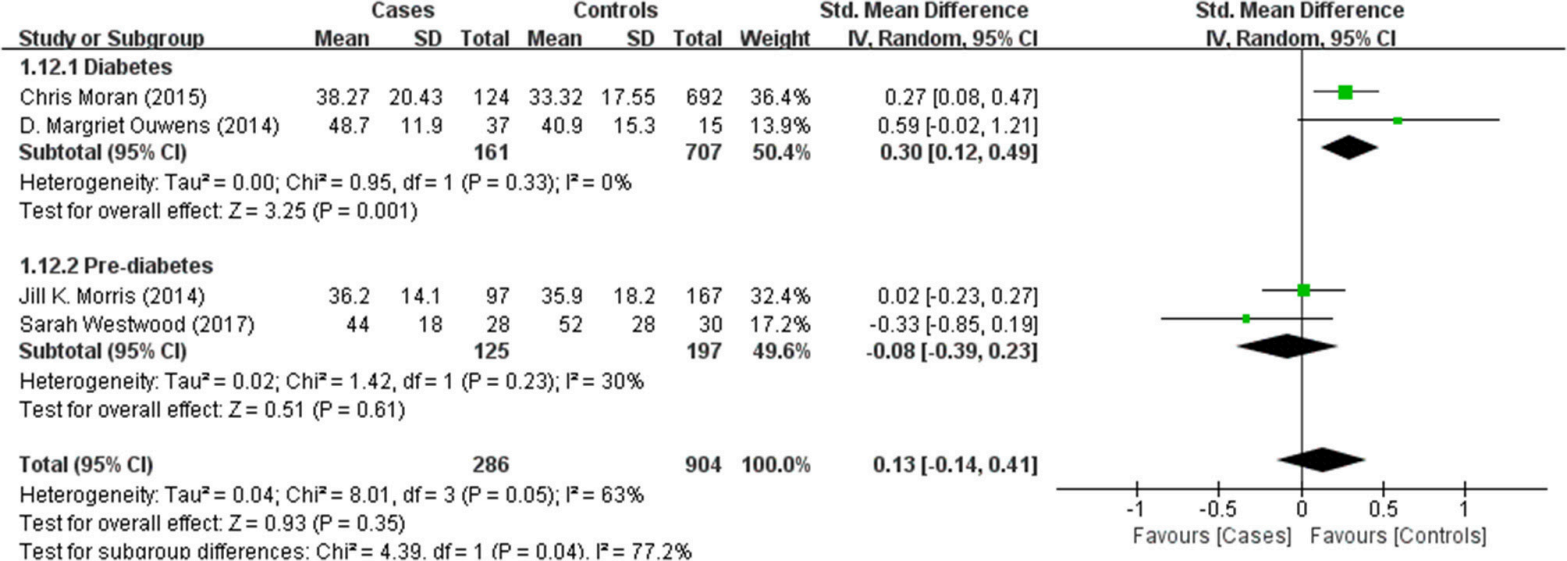
Subgroup analysis of CSF p-Tau levels in subjects with diabetes or pre-diabetes.

Results of subgroup analyses which explored potential sources of heterogeneity were summarized in Table 3. Pooled effects of Aβ1-42 and t-Tau in the CSF between cases and controls were significant in studies recruited through memory clinics (Aβ1-42: SMD: −1.76; 95% CI: −2.27, −1.25; *I*^2^ = 0%, *P* < 0.001; t-Tau: SMD: 1.15; 95% CI: 0.25, 2.05; I^2^ = 73%, *P* = 0.01) compared with nonsignificant correlations found from studies not recruited through memory clinics (Aβ1-42: SMD: 1.48; 95% CI: −0.85, 3.81; *I*^*2*^ = 100%, *P* = 0.21; t-Tau: SMD: −0.01; 95% CI: −0.47, 0.46; *I*^*2*^ = 71%, *P* = 0.98; see Figures 5, 6). Pooled effects of p-Tau in the CSF across studies that only included diabetic cases were significant (SMD: 0.30; 95% CI: 0.12, 0.49; *I*^*2*^ = 0%, *P* < 0.001) compared with nonsignificant correlations found from studies that only included pre-diabetic cases (SMD: −0.08; 95% CI: −0.39, 0.23; *I*^*2*^ = 30%, *P* = 0.21; see Figure 7). Both of which were affected by a substantial degree of heterogeneity. Neither mean age of groups nor quality score was significant moderators of the association between (pre)diabetes and AD-type CSF biomarkers (all subgroup analyses, *P* > 0.05; subgroup *I*^*2*^ range from 30 to 100%; see Table 3).

**Table 3:** Subgroup analyses of Aβ1-42, t-Tau, and p-Tau in the CSF between (pre)diabetic cases and controls.

**Outcomes**	**Subgroup**	**Studies (*n*)**	**Std. mean difference (95%CI)**	**I^2(%)^**	**P for heterogeneity**
**A**β**1-42**	**Total**	7	0.56 (−1.37, 2.49)	99	0.57
	**Cases**
	Diabetes	4	1.46 (−2.32, 5.23)	100	0.45
	Pre-diabetes	3	−0.59 (−1.52, 0.34)	91	0.22
	**Mean age**
	< 65 years	3	−0.29 (−1.46, 0.89)	91	0.63
	>65 years	4	2.08 (−0.76, 4.92)	100	0.15
	**Recruited through memory clinics**
	Yes	2	−1.76 (−2.27, −1.25)	0	< 0.001^*^
	No	5	1.48 (−0.85, 3.81)	100	0.21
	**Quality score**
	8	4	−0.31 (−1.07, 0.45)	90	0.42
	5–7	3	1.76 (−3.23, 6.75)	100	0.49
**t-Tau**	**Total**	5	0.39 (−0.15, 0.94)	84	0.15
	**Cases**
	Diabetes	3	0.10 (−0.50, 0.70)	80	0.74
	Pre-diabetes	2	0.91 (−0.46, 2.27)	90	0.19
	**Mean age**
	< 65 years	3	0.10 (−0.64, 0.83)	79	0.8
	>65 years	2	0.88 (−0.51, 2.26)	93	0.21
	**Recruited through memory clinics**
	Yes	2	1.15 (0.25, 2.05)	73	0.01^*^
	No	3	−0.01 (−0.47, 0.46)	71	0.98
	**Quality score**
	8	3	0.39 (−0.80, 1.58)	91	0.52
	5–7	2	0.36 (−0.08, 0.81)	54	0.11
**p-TAU**	**Total**	4	0.13 (−0.14, 0.41)	63	0.35
	**Cases**
	Diabetes	2	0.30 (0.12, 0.49)	0	0.001^*^
	Pre-diabetes	2	−0.08 (−0.39, 0.23)	30	0.61
	**Mean age**
	< 65 years	2	0.12 (−0.79, 1.02)	80	0.8
	>65 years	2	0.16 (−0.09, 0.41)	61	0.21
	**Quality score**
	8	3	0.06 (−0.36, 0.47)	61	0.78
	5-7	1	0.27 (0.08, 0.47)	–	0.005

* *P < 0.05*.

## Discussion

Our analysis showed no differences in Aβ1-42, t-Tau, or p-Tau levels in the CSF between (pre)diabetic and control subjects. Seemingly, neurodegenerations induced by diabetes may not be readily associated with changes in the biomarkers of Alzheimer's type pathology in the CSF.

Domain-specific cognitive impairment and cognitive dysfunction such as MCI, AD, and other types of dementia have recently been recognized to be common complications of diabetes (Neth and Craft, 2017). In addition, several studies have suggested that metabolic disorders associated with alterations of insulin homeostasis are risk factors for developing cognitive decline and even dementia. These disorders include IR, hyperinsulinemia, and diabetes, which are also attributed to impaired performance in several neuropsychological functions (Bitra et al., 2015). The molecular and cellular mechanisms underlying defective brain in (pre)diabetes have been investigated, and protein glycation and increased oxidative stress may probably be etiologic factors of AD (De Felice, 2013). In addition, there are evidences to suggest that AD and diabetes may share common signs of cerebral alterations (van Duinkerken et al., 2012a,b). Hyperinsulinemia and hyperglycemia seem to accelerate brain aging by inducing amyloid oligomerization and tau hyperphosphorylation, but the results are conflicting.

Amyloid plaque is a classical pathological AD biomarker. As the major protein component of amyloid plaque, Aβ is generated by a sequential cleavage of β- and γ-secretase from amyloid precursor protein (APP) (Faull et al., 2014). Consequently, being less soluble and more likely to aggregate than other forms, Aβ1-42 exists as the predominant form of amyloid deposited in neuritic plaques (Jovanovic et al., 2014). Plenty of evidence has demonstrated that CSF Aβ1-42 level is significantly lower in mild AD or MCI patients than normal aging individuals, thus becoming a useful pathological biomarker for AD (Frisoni et al., 2009). Although we did not find significant differences in CSF Aβ1-42 levels between (pre)diabetic and nondiabetic subjects from the overall analysis, the subgroup analysis revealed that the CSF level of Aβ1-42 was significantly lower in (pre)diabetic subjects than the controls when they were recruited from memory clinics with cognitive dysfunction, which matched the profile characteristics in patients with MCI or AD. It has been known that diabetes is associated with microvascular lesions, which can contribute to an increased permeability of blood brain barrier (BBB) and thus change the distribution of Aβ1-42 (Serlin et al., 2011; Xu et al., 2017). However, some included studies found no difference in the CSF Aβ1-42 levels between IR and non-IR or impaired glycemia (IG) and non-IG subjects. In contrast, some studies found increased CSF Aβ1-42 level in both types of diabetic patients. The subgroup analysis suggested that the cognitive dysfunction in (pre)diabetic subjects may not be due to disturbed CSF Aβ1-42 biomarker load, but diabetes or IR may accelerate the decrease of CSF Aβ1-42 level in subjects with cognitive impairment.

Hyperphosphorylation of tau protein is associated with increased intracellular NFT formation in AD (Blennow and Hampel, 2003). Recent studies have found that patients with T1DM showed increased CSF p-Tau (Ouwens et al., 2014). Meanwhile, T2DM was also associated with greater CSF t-Tau and p-Tau (Moran et al., 2015). Consequently, subgroup analysis found that CSF levels of t-Tau only significantly increased in (pre)diabetic patients with cognitive dysfunction and p-Tau in diabetic patients were significantly higher than controls. These observations fit with animal studies on streptozotocin-induced diabetic mouse model in which hyperphosphorylation of tau protein has been found in the cortex and hippocampus by histopathologic measures (Kim et al., 2009; Jung et al., 2013). Given that, scholars have proposed several pathways through which diabetes may contribute to increased p-Tau in the brain. An increased p-Tau in AD brain samples may be attributed to impaired neuronal glucose metabolism and a consequent reduced β-O-linkage of N-acetylglucosamine to tau (Liu et al., 2009). In addition, chronic hyperglycemia can increase levels of advanced glycation end products (AGEs), which may lead to protein cross-linking and promote stabilization of the paired helical filament tau (Münch et al., 2012). However, the significant differences of t-Tau were not replicated in subjects not recruited from memory clinics. In addition, the nonsignificant results of p-Tau in prediabetic cases and controls suggested that the association between prediabetes and CSF levels of p-Tau was attenuated.

Contrary to the subgroup analysis, postmortem human studies on diabetes with AD pathology have shown that the cerebral load of tau-related NFTs are either lower (Ahtiluoto et al., 2010) or similar (Thambisetty et al., 2013) between diabetic individuals and nondiabetic ones. This is consistent with the nonsignificant results of our overall analysis of p-Tau. Discrepancies among difference studies may largely be attributed to limited sample sizes with heterogeneity. Only four eligible studies with 286 cases and 904 controls measuring CSF p-Tau levels were included, and subgroup analysis of diabetes and age just included two studies in each group. However, the subgroup of age below 65 years old was presented with even higher heterogeneity, which indicated that age among the recruited prediabetes and diabetes should be considered as a potential contributing factor, but the limited studies may obscure some difference in those CSF biomarkers among young prediabetes versus aged ones. Subjects with cognitive dysfunction (e.g., MCI, AD) were also included even if these four studies were not recruited from memory clinics. In addition, the Honolulu-Asia Aging Study using rigorous phenotyping of diabetes has found greater risk of AD pathology in T2DM patients, but only among those carriers of APOE ε4 allele (Peila et al., 2002). Geijselaers et al. (2018) have also found that diabetes is associated with higher levels of insulin in the CSF, and this association is related to cognitive impairment and AD-type biomarkers in noncarriers of the APOE ε4 allele. Besides, diabetic patients usually manifest comorbidities, such as kidney disease. Similar to AD, the prevalence of diabetes and comorbidities is also higher in the elderly. As a result, the roles of comorbidities correlated with dementia in diabetic patients were also evaluated. Kuo et al. have found the hazard ratio (HR) for dementia in diabetic subjects rose from 1.45 in those without comorbidities to 1.50 in those with kidney comorbidities (Kuo et al., 2015). Sasaki et al. also reported the incidence of dementia was strongly related with kidney diseases independent of other vascular factors (Sasaki et al., 2011). Another meta-analysis including 54,779 subjects suggested that kidney disease was an independent risk factor for cognitive decline (Etgen et al., 2012). Therefore, the comorbidities of diabetic patients may also contribute to the heterogeneity of studies. However, the lack of corresponding database makes it impossible to exclude subjects with cognitive dysfunction. Stratifying these clinical studies into groups according to APOE ε4 genotype and taking comorbidities into consideration may partly explain some of the insignificant associations from our analyses.

Limitations of this meta-analysis should be considered. The included studies exploring the relationship between AD-type CSF biomarkers and (pre)diabetes had relatively small numbers of subjects. Besides, the diabetic individuals with collected CSF also included subjects with objective cognitive disturbances, MCI, AD, or other types of cognitive dysfunction (Exalto et al., 2010). That might hamper proper conclusions on the effects of (pre)diabetes on the levels of the biomarkers examined. Thus, caution should be given to generalize our findings for the population at large. Furthermore, details of duration of (pre)diabetes and effectiveness of glucose control were unavailable in most studies, which would have provided valuable clues for us to explore our hypotheses.

## Conclusion

In conclusion, the associations between CSF levels of AD-type biomarkers and (pre)diabetic conditions may be affected by cognitive function, phenotypes of diabetes, and other clinical characteristics. The relatively higher heterogeneity and limited sample size contributed to the nonsignificant differences of AD-type biomarkers between (pre)diabetic cases and controls. Thus, it remains to be investigated whether the CSF levels of AD-type biomarkers change over time as well as to what extent these biomarkers relate to or can predict cerebral compromise in (pre)diabetic patients.

## Author contributions

ML conceived and designed the study. YL and XJ conducted the systematic search, screened articles, and selected eligible articles. SL and XJ extracted information from eligible studies. YL performed the analyses and interpreted the results. All authors read and approved the final manuscript.

### Conflict of interest statement

The authors declare that the research was conducted in the absence of any commercial or financial relationships that could be construed as a potential conflict of interest. The reviewer YL and handling Editor declared their shared affiliation at the time of the review.
